# Radiation-induced eCIRP causes macrophage phagocytic dysfunction via mitochondrial impairment and ferroptosis

**DOI:** 10.3389/fimmu.2025.1719613

**Published:** 2025-11-20

**Authors:** Mian Zhou, Gaifeng Ma, Jingsong Li, Satoshi Yamaga, Max Brenner, Monowar Aziz, Ping Wang

**Affiliations:** 1 Center for Immunology and Inflammation, The Feinstein Institutes for Medical Research, Manhasset, NY, United States; 2 Departments of Surgery and Molecular Medicine, Zucker School of Medicine at Hofstra/Northwell, Manhasset, NY, United States

**Keywords:** ionizing radiation, macrophage, eCIRP, mitochondria, ferroptosis, phagocytosis

## Abstract

Ionizing radiation causes immune dysfunction, increasing susceptibility to infection and mortality. Extracellular cold-inducible RNA-binding protein (eCIRP) is released from cells during irradiation. This study investigates how radiation-induced eCIRP release causes macrophage phagocytic dysfunction via ferroptosis, with a focus on the role of mitochondrial dysfunction. Peritoneal macrophages were exposed to 10-Gy irradiation. eCIRP levels in the culture supernatants were assessed post-irradiation by ELISA. Ferroptosis was assessed by measuring lipid peroxidation and glutathione peroxidase 4 (GPX4) expression. Mitochondrial function was assessed using Mito stress assay in a Seahorse metabolic analyzer. Phagocytic activity was quantified by measuring the uptake of pHrodo-labeled *E. coli*. Our results demonstrated that 10-Gy irradiation induced ferroptosis in peritoneal macrophages. Markers of ferroptosis, lipid peroxidation, were significantly elevated, and GPX4 was significantly downregulated in a time-dependent manner on days 3 and 5 post-irradiation. We unveiled a strong time-dependent correlation between post-irradiation eCIRP release and the increases in ferroptosis and macrophage phagocytic dysfunction at days 3 and 5. Furthermore, radiation-induced eCIRP positively correlated with mitochondrial dysfunction, evidenced by marked reductions in basal and maximal respiration and ATP production, mirroring effects of direct eCIRP treatment. Crucially, the application of MFG-E8-derived oligopeptide 3 (MOP3), a novel opsonic eCIRP inhibitor, effectively cleared eCIRP, restoring mitochondrial function, reducing ferroptosis, and improving phagocytosis in irradiated macrophages. These findings establish that radiation-induced eCIRP release drives mitochondrial dysfunction and ferroptosis, thereby impairing macrophage phagocytosis. Targeting eCIRP offers a promising therapeutic strategy to enhance host defense following radiation exposure.

## Introduction

Radiation exposure, whether from terrorist attacks, large-scale conflict nuclear events, accidents at nuclear power facilities or medical procedures like cancer radiotherapy raises serious concerns about radiation injury (1–4). High-dose radiation exposure induces acute radiation syndrome (ARS), characterized by various clinical complications, most notably hematopoietic dysfunction (5, 6). Damage to hematopoietic stem and progenitor cells leads to cytopenia (anemia, neutropenia, and thrombocytopenia), often accompanied by multiple organ dysfunction, ultimately leading to death (5, 6). Macrophage phagocytic activity is a critical immune function, essential for host defense against pathogens and for maintaining tissue homeostasis (7). Therefore, we focused on phagocytosis as a pivotal functional readout to assess macrophage activity following radiation exposure. While radiation-induced neutropenia increases infection susceptibility, the relative radioresistance of macrophages underscores their critical role in host defense following radiation injury (3, 5, 8). However, the direct effects of radiation on macrophage function remain poorly understood (8). Therefore, it is crucial to elucidate how radiation impacts macrophage survival, viability, and phagocytic function.

Ferroptosis, an iron-dependent form of regulated cell death characterized by lipid peroxidation, was first described in 2012 (9). It has been implicated in various pathologies, including post-radiation cell death in multiple organ systems (4, 10). Therapeutic doses of radiation can polarize macrophages towards M1 or M2 phenotypes, altering their immune activity (8, 11). However, it is unknown whether radiation induces macrophage ferroptosis. This study aims to investigate the role of ferroptosis in radiation-induced macrophage dysfunction and explore its potential as a therapeutic target.

Mitochondria are central to cellular metabolic reprogramming and regulate various forms of cell death, including ferroptosis (12, 13). Mitochondrial dysfunction can lead to the accumulation of free iron and reactive oxygen species (ROS), promoting ferroptosis (12). Extracellular cold-inducible RNA-binding protein (eCIRP), a novel damage-associated molecular pattern (DAMP), is released after radiation exposure (14). eCIRP induces endoplasmic reticulum stress and ROS production plays a significant role in sepsis-associated acute lung injury (15, 16). eCIRP also links to ischemia-reperfusion associated organ injuries (16). We observed a correlation between post-irradiation eCIRP levels and the severity of mitochondrial dysfunction in macrophage. Among several eCIRP antagonists, a new opsonic inhibitor, named milk fat globule-EGF-factor VIII (MFG-E8)-derived small opsonic peptide 3 (MOP3), has been discovered to selectively target eCIRP and facilitate its phagocytic clearance in an αvβ3-integrin-dependent manner. Studies have shown that MOP3 clears eCIRP, attenuates inflammation *in vitro* and *in vivo*, and protects the host from sepsis and intestinal ischemia-reperfusion injury (17, 18). The role of MOP3 in radiation injury in terms of scavenging eCIRP to mitigate eCIRP-induced effects has not been studied.

Therefore, this study investigated the impact of radiation-induced eCIRP release on mitochondrial dysfunction and its link to macrophage ferroptosis and phagocytic dysfunction. We also aimed to study the effects of MOP3 on restoring macrophage phagocytic function by mitigating ferroptosis and correcting macrophage metabolic dysfunction. Our findings on the role of eCIRP in mitochondrial injury and radiation-induced macrophage ferroptosis and phagocytic dysfunction reveal the mechanism and potential therapeutic strategy using MOP3 for mitigating radiation injury.

## Materials and methods

### Animals

Male C57BL/6 mice (8–12 weeks old) from Jackson Laboratory (Bar Harbor, ME, USA) were housed under standard laboratory conditions (12-hour light/dark cycle, free access to food and water). All animal experiments were performed in accordance with NIH guidelines and approved by the Institutional Animal Care and Use Committee of the Feinstein Institutes for Medical Research (ethical approval number: 2024-1093).

### Cell culture, radiation exposure and MOP3 treatments

Peritoneal macrophages (PerM) from C57BL/6 mice were harvested following CO_2_ asphyxiation by washing peritoneal cavities with cold PBS containing 2% FBS (14). Collected cells were centrifuged (300 x g, 10 minutes, 4 °C), washed with PBS, and cultured in RPMI 1640 supplemented with 10% heat-inactivated FBS, 2 mM glutamine, 1% penicillin-streptomycin, and 10 mM HEPES. Non-adherent cells were removed after 3 hours incubation. After overnight culture, adherent PerM were irradiated with 10 Gy (3 Gy/min) using an X-ray Precision X-Rad320 (Precision X-Ray Inc.). Macrophages are relatively radioresistant compared to other immune cells. A radiation dose of 10-Gy was selected based on our preliminary experiments and a previous study (14). While primarily focused on identifying countermeasures for severe radiation injuries resulting from high-dose exposures (e.g., terrorist attacks or nuclear facility accidents), our findings also hold relevance for understanding the cumulative effects of lower-dose radiation exposure during cancer radiotherapy. Ferrostatin-1 (Fer-1, 2 µM, HY-100579, MedChemExpress) or vehicle was administered at 2 hours post-irradiation. In an additional group of macrophages, milk fat globule-epidermal growth factor-factor VIII (MFG-E8)-derived oligopeptide-3 (MOP3; 10 µg/ml, Synthesized by Genscript) or vehicle was added at 5- and 24-hours post-irradiation. MOP3 was discovered and developed in our previous study (17). In brief, theoretical analyses demonstrated the strongest binding between eCIRP and discoidin domain-2 (DD-2) of MFG-E8 (17). The known amino acid (AA) sequence of DD-2 from murine MFG-E8 was split into sequences approximately 15-AAs in length, designed to bind to eCIRP. These 15-AA sequences were then tagged with a 3-AA-long sequence, Arg-Gly-Asp (RGD), designed to bind to the integrin receptor. The resulting peptides were screened *in vitro* for their ability to attenuate eCIRP-induced TNFα production (17). The selected peptides underwent further testing by flow cytometry and Biacore analyses to confirm their ability to link eCIRP to the α_v_β_3_-integrin receptor, resulting in greater clearance of eCIRP. These experiments yielded MOP3 with amino acid sequence: RGDSSSYKTWNLRAFGWY (17). Cell viability, toxicity, lipid peroxidation, mitochondrial function, and bacterial phagocytosis were assessed at 3- and 5-day post-irradiation.

RAW264.7 cells (ATCC) were cultured in complete DMEM supplemented with 10% heat-inactivated FBS, 2 mM glutamine, 1% penicillin-streptomycin. The cells were treated with rmCIRP/eCIRP (0.1 µg/ml and 1.0 µg/ml) for 16 hours before mitochondrial function assessment.

### Assessment of eCIRP by ELISA

Peritoneal macrophages were collected from mice and irradiated with a single dose of 10-Gy. At 3- and 5-day post-irradiation, cell culture supernatants from irradiated cells were collected and eCIRP levels were measured using an ELISA kit (EM6504, American Research Products, Waltham, MA) following the instruction provided by the manufacture. Recombinant mouse CIRP was used as standard to quantify the levels of eCIRP. Briefly, 100 µL samples or CIRP standard were loaded into each well in the plate that was pre-coated with anti-CIRP antibody. Then, the plate was incubated for 90 min at 37°C followed by washing with buffer. Biotin-labeled anti-CIRP antibody was then added to the plate and incubated for 60 min at 37°C. After washing, HRP-Streptavidin conjugated secondary antibody was added and incubated for 30 min at 37°C. After reaction with substrate, the plate was read using a BioTek Synergy Neo2 multimode reader (BioTek) at 450 nm.

### Cell viability and cell toxicity assay

Cell viability was measured using the CellTiter 96 AQ non-radioactive cell proliferation assay kit (G5421, Promega). This assay quantifies the reduction of MTS to formazan by metabolically active cells, with absorbance measured at 490 nm. Decreased formazan production indicates reduced cell viability. Cytotoxicity was assessed using the CyQUANT lactate dehydrogenase (LDH) Cytotoxicity Assay Kit (C20301, Invitrogen). This assay measures the release of LDH, a cytosolic enzyme, into the culture medium, which is indicative of plasma membrane damage and cellular injury.

### Lipid peroxidation assay

Lipid peroxidation in the cell was measured using Image-it lipid peroxidation kit (C10445, Life Technologies). BODIPY 581/591 C11 reagent is a sensitive fluorescent reporter for lipid peroxidation. According to the instructions from the manufacture, the BODIPY reagent was added into the cell culture medium. Upon oxidation in live cells, fluorescence shifts from red to green, providing a ratio metric indication of lipid peroxidation. The fluorescent intensity was measured using BioTek Synergy Neo2 multimode reader (BioTek) and the fluorescent images were taken by Evos cell imaging system (Thermo Fisher Scientific).

### Mitochondria function measurement

Mitochondrial function in peritoneal macrophages was assessed using the Mito Stress Test Kit (103015-100, Agilent) and a Seahorse XF Pro Live-Cell Metabolic Analyzer (Agilent). PerM (1 × 10^5^ cells/well) were seeded in Seahorse microplates, irradiated with 10 Gy, and analyzed at 3- and 5-day post-irradiation. The assay protocol involved sequential injections of oligomycin (1.5 µM), carbonyl cyanide-4 (trifluoromethoxy) phenylhydrazone (FCCP), and a mixture of rotenone and antimycin A. Oligomycin, an ATP synthase inhibitor, was used to determine mitochondrial ATP production. FCCP, an uncoupler that collapses the proton gradient and disrupts the mitochondrial membrane potential, was used to assess maximal respiration and spare respiratory capacity. Rotenone and antimycin A, inhibitors of complexes I and II respectively, were used to determine non-mitochondrial respiration. Mitochondrial function was also assessed in macrophages treated with recombinant murine CIRP (rmCIRP/eCIRP, synthesized by CUSABIO Technology).

### Bacterial phagocytosis assay

Peritoneal macrophages were collected from mice and seeded in 96-well black tissue culture plates with transparent bottoms (3603, Corning Life Sciences). Following 10-Gy irradiation, phagocytosis was assessed at 3- and 5-days post-irradiation. Opsonized pHrodo™ Green *E. coli* (3 × 10^7^/well, P35366, Thermo Fisher Scientific) were added to each well and incubated for 1.5 hours at 37°C. Phagocytosis was quantified by measuring the fluorescence of internalized bacteria (Ex/Em 490/533 nm) using a BioTek Synergy Neo2 multimode reader (BioTek). Cells were then fixed with 4% paraformaldehyde, and fluorescent images were acquired.

### Western blotting

Peritoneal macrophages were collected from mice and irradiated with a single dose of 10 Gy. At 3- and 5-days post-irradiation, cells were harvested and lysed in lysis buffer (10 mM Tris-HCl [pH 7.5], 100 mM NaCl, 1 mM EDTA, 1 mM EGTA, 1% Triton X-100) supplemented with protease inhibitor cocktails (PI88665, Thermo Fisher Scientific). Protein concentrations were determined using the DC protein assay kit (500-0112, Bio-Rad). Samples were separated by SDS-PAGE on 4-12% gradient gels and transferred to nitrocellulose membranes. Membranes were blocked with 0.1% casein in Tris-buffered saline for 1 hour at room temperature and incubated overnight at 4°C with primary antibodies against GPX4 (67763-1-Ig, Proteintech, 1:300), Rac1 (4651S, Cell Signaling Technology, 1:300), and β-actin (A5441, MilliporeSigma, 1:30,000). After washing, membranes were incubated with infrared dye-labeled secondary antibodies (Li-Cor Biosciences). Protein bands were visualized using the Odyssey CLx imaging system and quantified using Image Studio 5.2 software (Li-Cor Biosciences).

### Statistical analysis

GraphPad Prism software was used to analysis the data. Data were analyzed for normality and are presented as mean ± standard error (SE). One-way ANOVA with Tukey’s test was performed to compare multiple groups. An unpaired two tailed Student’s t test was used for 2-group comparisons. Statistical significance was set at p < 0.05.

## Results

### Radiation increases eCIRP release and impairs macrophage phagocytic function

**Figure 1:**
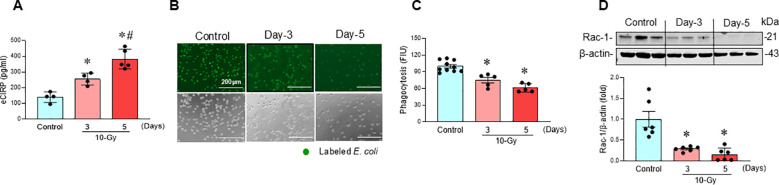
Radiation-induced eCIRP release causes macrophage phagocytic dysfunction. Peritoneal macrophages were irradiated with a single dose of 10-Gy and cells were analyzed on day-3 and day-5 post-irradiation. **(A)** The released eCIRP in the culture medium was measured by ELISA. Data are expressed as the mean ± SE (n = 4-5/group). The groups were compared by one-way ANOVA and the Tukey’s test. *p < 0.05 vs. non-radiation control; ^#^*p* < 0.05 vs. day-3 post-irradiation. **(B, C)** Macrophage phagocytosis was determined by their engulfment of PHrodo labeled *E. coli.* The representative images are shown, and fluorescent intensity obtained from the plate reader was demonstrated as bar diagram. Data are expressed as the mean ± SE (n = 5-10/group). The groups were compared by one-way ANOVA and the Tukey’s test. *p < 0.05 vs. non-radiation control. The experiments were performed 2 times. **(D)** Rac-1 protein expression was assessed by western blotting. Representative blots and the corresponding bar diagram are shown. Full uncropped gel images are shown in **Supplementary Materials**. The experiment was performed three times. The data presented are expressed as the mean ± SE (n = 6/group). The control was set to 1 for normalization. The groups were compared by one-way ANOVA and the Tukey’s test. *p < 0.05 vs. the non-radiation control.

Following 10-Gy X-ray irradiation of peritoneal macrophages, the release of eCIRP was assessed at different time points post-irradiation. Irradiated macrophages exhibited a significant time-dependent increase in eCIRP levels in the culture supernatant, with the highest release observed on day-5 post-irradiation compared to day-3 post-radiation (**Figure 1A**). We also determined elevated eCIRP levels at an earlier time point (day-2) following radiation exposure (**Supplementary Figure 2**). Given that eCIRP was most notably released on day-3 and day-5, we focused our subsequent analysis on day-3 and day-5. This rise in eCIRP levels correlated with a reduction in macrophage phagocytic activity (**Figures 1B, C**). Furthermore, Rac1, a Rho GTPase critical for phagocytosis, was significantly downregulated in a time-dependent manner following irradiation (**Figure 1D**). This data suggests that irradiation induces eCIRP release from macrophages, which contribute to macrophage impaired phagocytic function.

### Radiation induces macrophage ferroptosis

**Figure 2:**
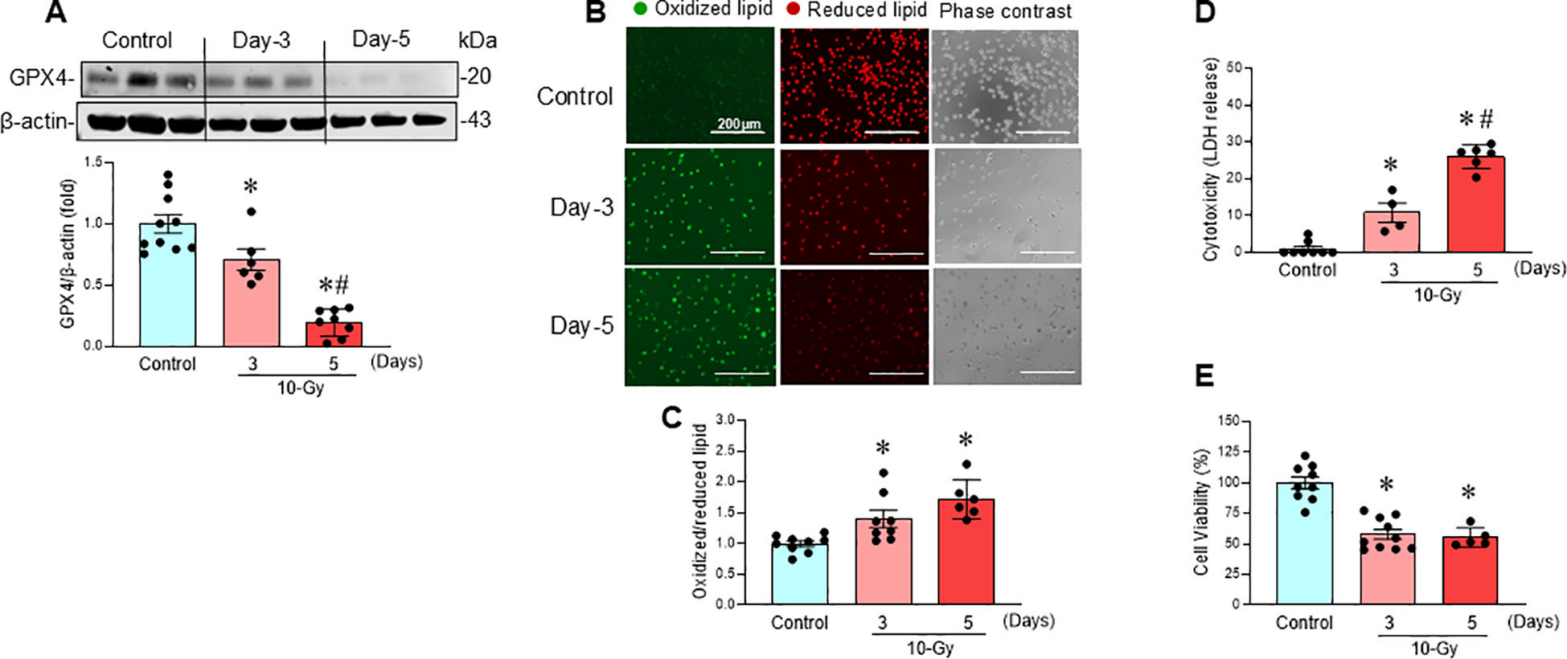
Radiation induces macrophage ferroptosis. Peritoneal macrophages were irradiated with a single dose of 10-Gy and analyzed on day-3 and day-5 post-irradiation. **(A)** GPX4 protein expression was assessed by Western blotting. Representative blots are shown. Full uncropped gel images are shown in **Supplementary Materials**. Data are expressed as the mean ± SE (n = 6–10/group). The non-radiation control was set to 1 for normalization. The experiments were performed 3 times. The groups were compared by one-way ANOVA and the Tukey’s test. *p < 0.05 vs. non-radiation control; ^#^*p* < 0.05 vs. day-3 post-irradiation. **(B)** Lipid peroxidation was assessed by a sensitive fluorescent reporter. Reduced form of lipid shows red and peroxidized lipid shows green fluorescence as indicated in the images. **(C)** The fluorescence of oxidized/reduced ratio was calculated, and non-radiation control was set to 1 for normalization. Data are expressed as the mean ± SE (n = 5-9/group). The groups were compared by one-way ANOVA and the Tukey’s test. *p < 0.05 vs. non-radiation control. The experiments were performed 2 times. **(D)** Cytotoxicity was evaluated by LDH release. Non-radiation control was set to 1 for normalization. Data are expressed as the mean ± SE (n = 4-8/group). The groups were compared by one-way ANOVA and the Tukey’s test. *p < 0.05 vs. non-radiation control; ^#^*p* < 0.05 vs. day 3 post-irradiation. The experiments were performed 2 times. **(E)** Cell viability was determined by MTS reaction. Non-radiation control was set to 100 for normalization. Data are expressed as the mean ± SE (n = 5-10/group). The groups were compared by one-way ANOVA and the Tukey’s test. *p < 0.05 vs. non-radiation control. The experiments were performed twice independently.

Following 10-Gy X-ray irradiation of peritoneal macrophages, ferroptosis, a form of regulated cell death, was assessed at various time points post-irradiation. GPX4, a key negative regulator of ferroptosis that reduces lipid hydroperoxides, was significantly downregulated after irradiation, decreasing by 29% on Day 3 and 80% on Day 5 (**Figure 2A**). Concurrently, lipid peroxidation, a hallmark of ferroptosis, increased significantly by 49% and 58% on Days 3 and 5 post-irradiation, respectively (**Figures 2B, C**). Further evidence of ferroptosis post-irradiation was the significant increase in LDH release, indicating cytotoxicity (**Figure 2D**), and the significant decrease in MTS activity, indicating reduced cell viability (**Figure 2E**). These data suggest that irradiation induces ferroptosis in macrophages.

### Radiation-induced eCIRP causes mitochondrial dysfunction

**Figure 3:**
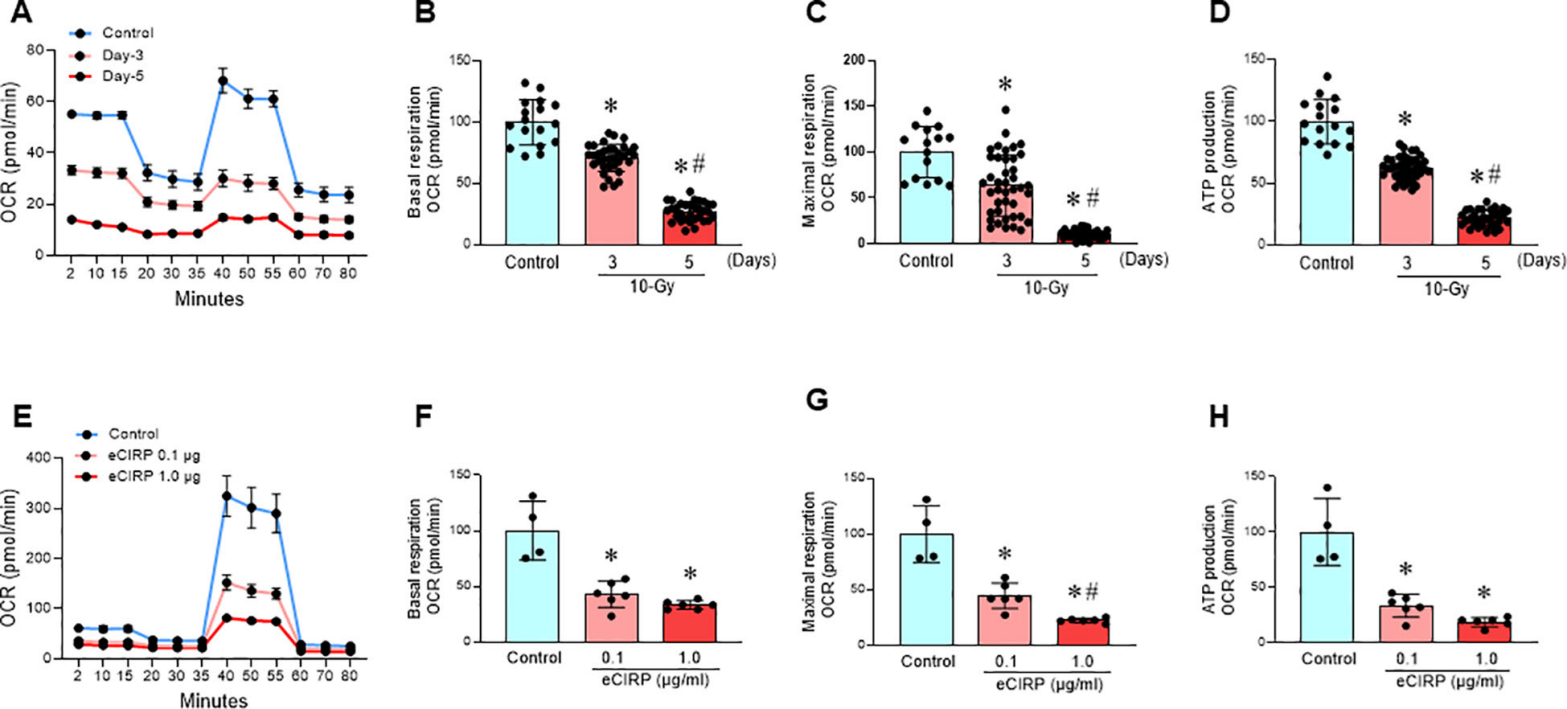
Radiation-induced eCIRP release causes mitochondrial dysfunction. **(A-D)** Peritoneal macrophages were irradiated with a single dose of 10-Gy and mitochondria function were assessed on day-3 and day-5 post-irradiation using Mito Stress Assay in a Seahorse XF Analyzer. **(A)** The real-time measurement of cellular oxygen consumption rate (OCR) indicated as kinetics is shown. **(B-D)** Basal and maximal respiration and ATP production are shown in bar diagrams. The data are expressed as the mean ± SE (n = 16-47/group). The non-radiation control was set to 100 for normalization. The groups were compared by one-way ANOVA and the Tukey’s test. *p < 0.05 vs. the non-radiation control; ^#^*p* < 0.05 vs. day 3 post-irradiation. The experiments were performed two times. **(E-H)** RAW264.7 macrophages were treated with rmCIRP (0.1 and 1.0 µg/ml) for 16 h and mitochondria function was assessed using Mito Stress Assay in a Seahorse XF Analyzer. **(E)** The real-time measurement of cellular oxygen consumption rate (OCR) indicated as kinetic is shown. **(F-H)** Basal and maximal respiration and ATP production are shown in bar diagrams. The data are expressed as the mean ± SE (n = 4-6/group). The control was set to 100 for normalization. The groups were compared by one-way ANOVA and the Tukey’s test. *p < 0.05 vs. control group; ^#^*p* < 0.05 vs. 0.1 µg/ml rmCIRP.

Mitochondria, the primary organelles of cellular respiration and ATP production, are essential for cellular functions (19). Mitochondrial damage, often linked to increased reactive oxygen species (ROS) production, impair various cellular processes, contribute to ferroptosis (13, 20). Following 10-Gy irradiation of peritoneal macrophages, both basal and maximal mitochondrial respiration (indicated as oxygen consumption rate, OCR) were significantly reduced, by 29% and 37%, respectively, at Day 3 post-irradiation (**Figures 3A–C**). These reductions were further exacerbated on Day 5 post-irradiation, reaching 73% and 90%, respectively (**Figures 3A–C**). Mitochondrial ATP production also decreased significantly, by 38% on Day 3 and 78% on Day 5 post-irradiation (**Figure 3D**). This time-dependent decline in mitochondrial function strongly correlated with the observed increase in eCIRP levels (**Figure 1A**), suggesting a potential role for eCIRP in radiation-induced mitochondrial dysfunction.

To further investigate the role of eCIRP in mitochondrial dysfunction, mouse macrophages cell line, RAW264.7 cells were treated with eCIRP (0.1 and 1.0 µg/ml) for 16 h. Both concentrations significantly reduced basal and maximal mitochondrial respiration and inhibited mitochondrial ATP production by 67% and 81%, respectively (**Figures 3E–H**). These findings strongly implicate eCIRP in radiation-induced mitochondrial dysfunction. eCIRP impairs mitochondria function also confirmed in PerM (**Supplementary Figures 1A-D**).

### MOP3 corrects mitochondrial dysfunction, reduces ferroptosis and improves macrophage phagocytosis after irradiation

**Figure 4:**
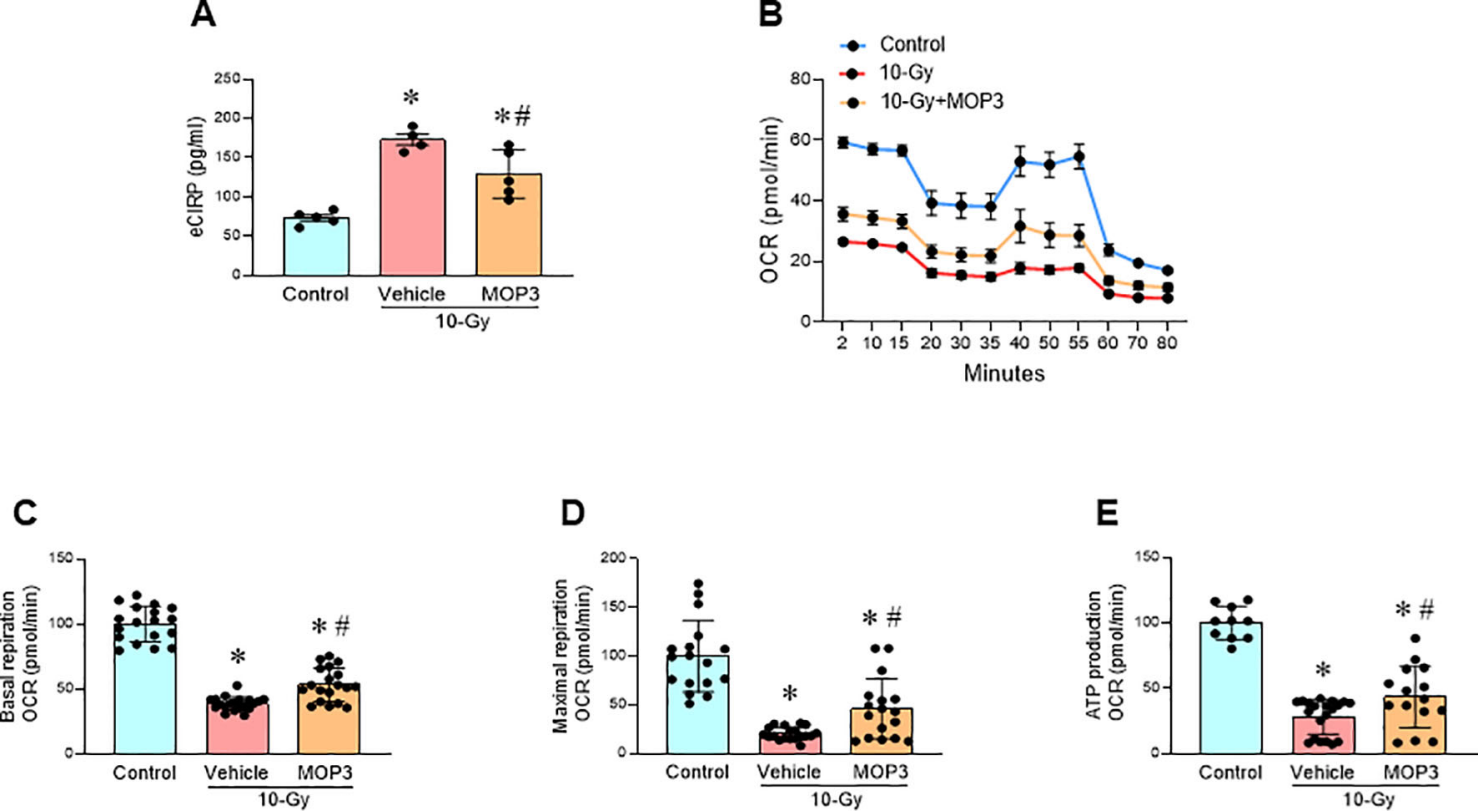
eCIRP clearance mitigates mitochondrial dysfunction after radiation. Peritoneal macrophages were irradiated with a single dose of 10-Gy and treated with MOP3 (10 µg/ml) or vehicle at 5 and 24 h post-irradiation. The cells were analyzed on day 3 post-irradiation. **(A)** eCIRP levels in the culture medium were measured by ELISA. The data are expressed as the mean ± SE (n = 4-5/group). The groups were compared by one-way ANOVA and the Tukey’s test. *p < 0.05 vs. non-radiation control; ^#^*p* < 0.05 vs. 10-Gy with vehicle treatment. **(B)** Mitochondria function was assessed using Mito Stress Assay in a Seahorse XF Analyzer. The real-time measurement of cellular oxygen consumption rate (OCR) indicated as kinetic is shown. **(C-E)** Basal and maximal respiration and ATP production are shown in bar diagrams. The data are expressed as the mean ± SE (n = 10-20/group). The non-radiation control was set to 100 for normalization. The groups were compared by one-way ANOVA and the Tukey’s test. *p < 0.05 vs. non-radiation control; ^#^*p* < 0.05 vs. 10-Gy with vehicle treatment.

**Figure 5:**
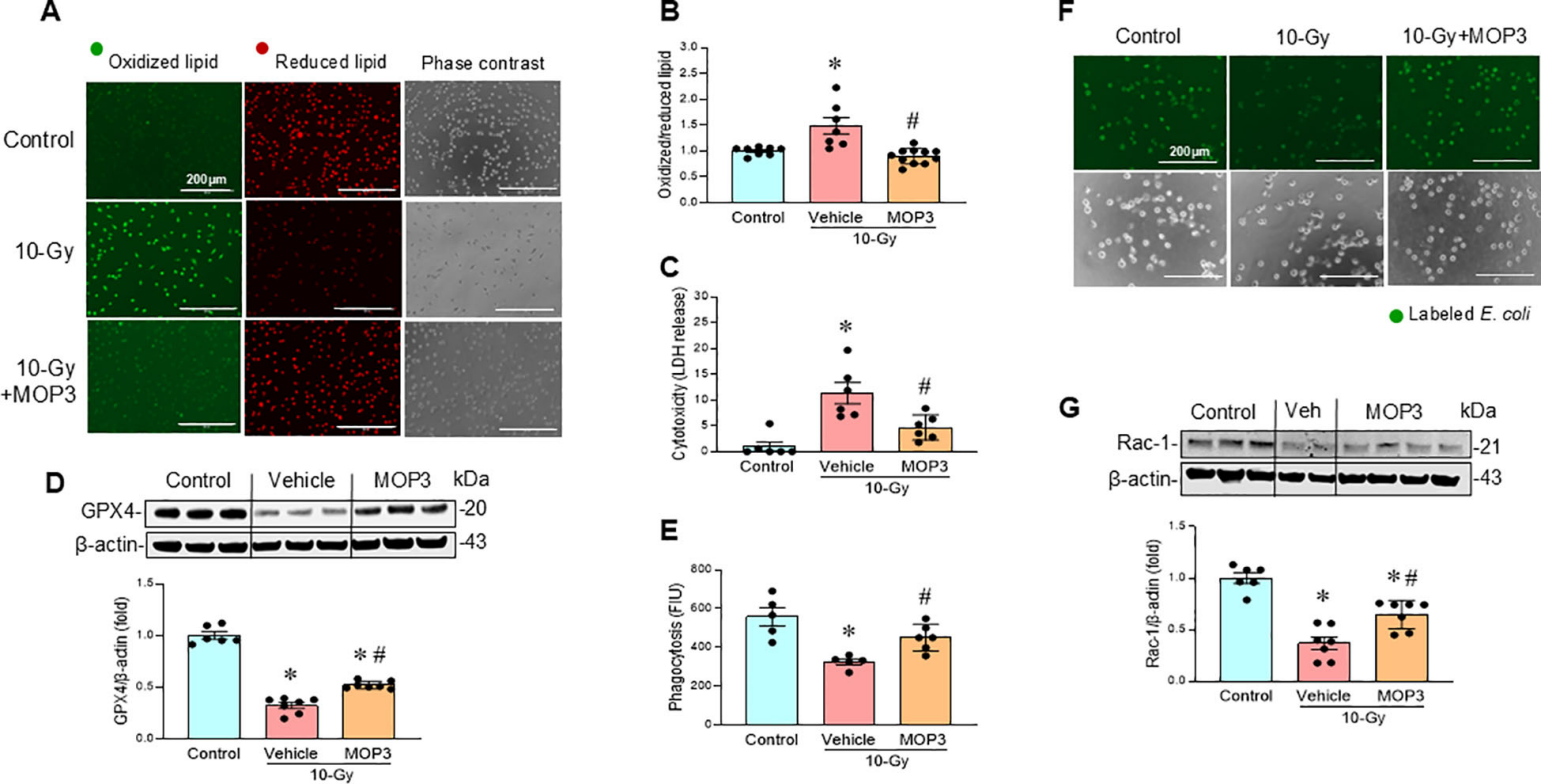
eCIRP clearance reduces ferroptosis and improves macrophage phagocytosis after irradiation. Peritoneal macrophages were irradiated with a single dose of 10-Gy and treated with MOP3 (10 µg/ml) or vehicle at 5 and 24 h post-irradiation. The cells were analyzed on day 3 post-irradiation. **(A)** Lipid peroxidation was assessed by a sensitive fluorescent reporter. Reduced form of lipid shows red and peroxidized lipid shows green fluorescence as indicated in the images. **(B)** The fluorescence of oxidized/reduced ratio was calculated and showed in the bar diagram. Non-radiation control was set to 1 for normalization. Data are expressed as the mean ± SE (n = 7-11/group). The groups were compared by one-way ANOVA and the Tukey’s test. *p < 0.05 vs. non-radiation control; *^#^p* < 0.05 vs. 10-Gy with vehicle treatment. The experiments were performed 2 times. **(C)** Cytotoxicity was evaluated by LDH release. Non-radiation control was set to 1 for normalization. Data are expressed as the mean ± SE (n = 6/group). The groups were compared by one-way ANOVA and the Tukey’s test. *p < 0.05 vs. non-radiation control; ^#^*p* < 0.05 vs. 10-Gy with vehicle treatment. The experiments were performed 2 times. **(D)** GPX4 protein levels were assessed by western blotting. Representative blots and the corresponding bar diagram are shown. Full uncropped gel images are shown in **Supplementary Materials**. The experiment was performed two times. The data presented were obtained from two independent experiments and are expressed as the mean ± SE (n = 6-7/group). The control was set to 1 for normalization. The groups were compared by one-way ANOVA and the Tukey’s test. *p < 0.05 vs. the non-radiation control; ^#^*p* < 0.05 vs. 10-Gy with vehicle treatment. **(E-G)** Macrophage phagocytosis was determined by their engulfment of PHrodo labeled *E. coli.* and Rac1 status **(E, F)**. The green fluorescence from the internalized *E. coli.* is shown in the representative images. The fluorescent intensity from engulfed *E. coli* was obtained from the plate reader and shown in the bar diagram. Data are expressed as the mean ± SE (n = 4-6/group). The groups were compared by one-way ANOVA and the Tukey’s test. *p < 0.05 vs. non-radiation control; ^#^*p* < 0.05 vs. 10-Gy with vehicle treatment. **(G)** Rac-1 protein expression was assessed by western blotting. Representative blots and the corresponding bar diagram are shown. Full uncropped gel images are shown in **Supplementary Materials**. The experiment was performed three times. The data presented were obtained from two independent experiments and are expressed as the mean ± SE (n = 6-7/group). The control was set to 1 for normalization. The groups were compared by one-way ANOVA and the Tukey’s test. *p < 0.05 vs. the non-radiation control; ^#^*p* < 0.05 vs. 10-Gy with vehicle treatment.

MOP3, a 15-AA peptide with binding to α_v_β_3_-integrin, was developed based on its strong binding to eCIRP (17). MOP3 binds eCIRP to facilitate α_v_β_3_-integrin-dependent internalization and lysosome-dependent degradation of MFG-E8/eCIRP complexes, resulting in greater clearance of eCIRP (17). MOP3 has been shown to attenuate sepsis and gut ischemia-reperfusion injury by opsonizing eCIRP and promoting its clearance (17, 18). In this study, following 10-Gy irradiation of peritoneal macrophages, MOP3 (10 µg/ml) was added at 5 h and 24 h post-irradiation. Mitochondrial function was assessed at Day 3 post-irradiation. The treatment of irradiated macrophages with MOP3 statistically decreased eCIRP levels in the culture supernatant (**Figure 4A**) and improved mitochondrial function post-irradiation (**Figures 4B–E**). Furthermore, MOP3 attenuated radiation-induced lipid peroxidation, LDH release, and inhibited the decrease of GPX4 levels in macrophages (**Figures 5A–D**), suggesting a reduction in ferroptosis. Irradiated macrophage phagocytic function was significantly improved by MOP3 treatment as compared to vehicle treated cells, which was supported by the increase of Rac-1 protein levels with MOP3 treatment (**Figures 5E–G**). Collectively, eCIRP clearance reduced mitochondria dysfunction, ferroptosis and improved macrophage phagocytosis post irradiation.

### Ferroptosis inhibition restores macrophage phagocytic function

**Figure 6:**
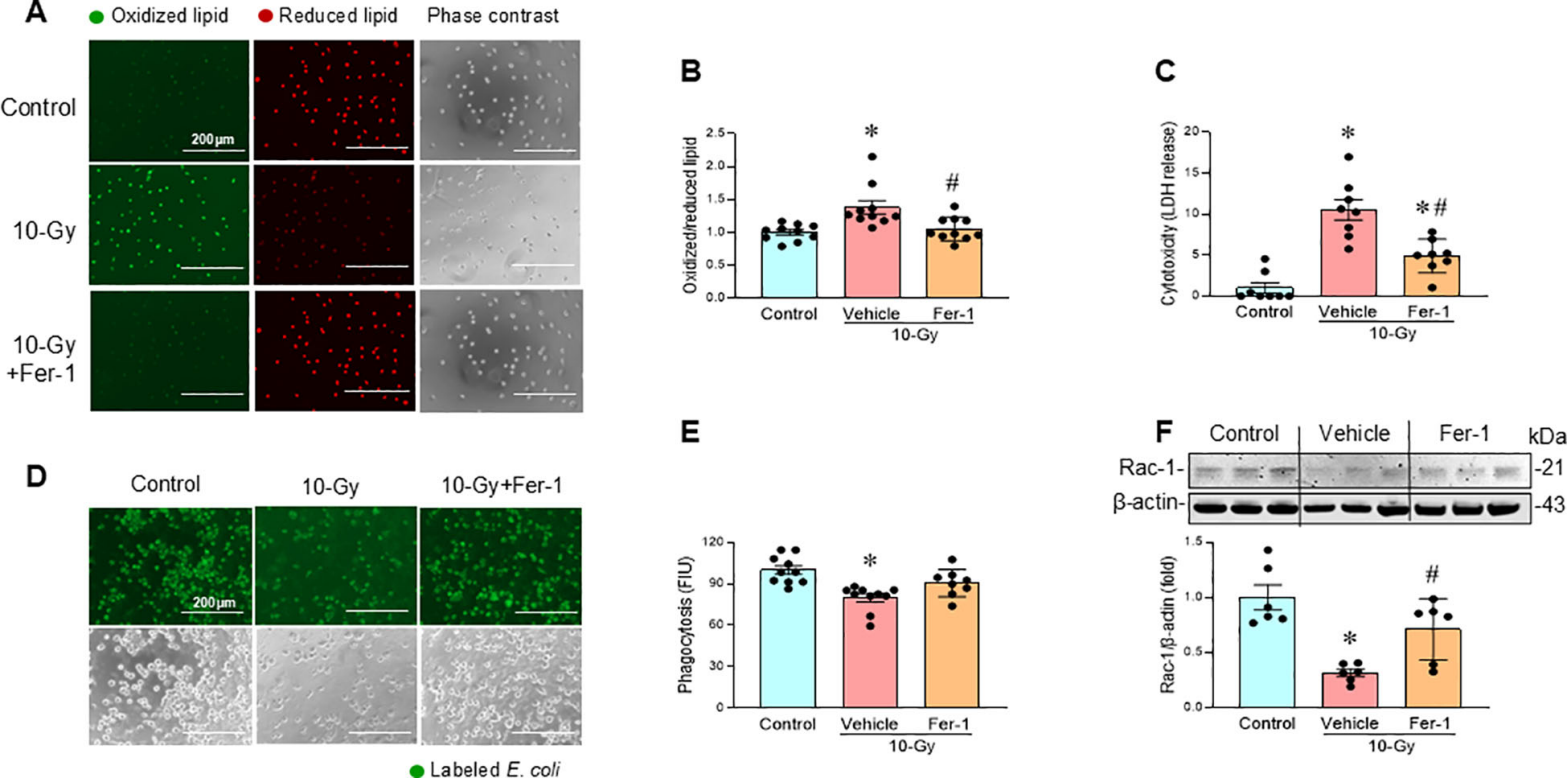
Pharmacologic inhibition of ferroptosis restores macrophage phagocytosis. Peritoneal macrophages were irradiated with a single dose of 10-Gy and treated with Fer-1 (2 µM) or vehicle at 2 h post-irradiation. The cells were analyzed on day-3 post-irradiation. **(A, B)** Lipid peroxidation was assessed by a sensitive fluorescent reporter. Reduced form of lipid shows red and peroxidized lipid shows green fluorescence as indicated in the images. The fluorescence of oxidized/reduced ratio was calculated and showed in the bar diagram. Non-radiation control was set to 1 for normalization. Data are expressed as the mean ± SE (n = 10/group). The groups were compared by one-way ANOVA and the Tukey’s test. *p < 0.05 vs. non-radiation control; ^#^*p* < 0.05 vs. 10-Gy with vehicle treatment. The experiments were performed two times. **(C)** Cytotoxicity was evaluated by LDH release. Non-radiation control was set to 1 for normalization. Data are expressed as the mean ± SE (n = 8/group). The groups were compared by one-way ANOVA and the Tukey’s test. *p < 0.05 vs. non-radiation control; ^#^*p* < 0.05 vs. 10-Gy with vehicle treatment. The experiments were performed 2 times. **(D, E)** Macrophage phagocytosis was determined by their engulfment of PHrodo labeled *E. coli.* The green fluorescence from the internalized *E. coli.* is shown in the representative images. The fluorescent intensity from engulfed *E. coli* was obtained from the plate reader and shown in the bar diagram. Data are expressed as the mean ± SE (n = 8-10/group). The groups were compared by one-way ANOVA and the Tukey’s test. *p < 0.05 vs. non-radiation control. **(F)** Rac-1 protein expression was assessed by western blotting. Representative blots and the corresponding bar diagram are shown. Full uncropped gel images are shown in **Supplementary Materials**. The data are expressed as the mean ± SE (n = 6/group). The control was set to 1 for normalization. The groups were compared by one-way ANOVA and the Tukey’s test. *p < 0.05 vs. the non-radiation control; ^#^*p* < 0.05 vs. 10-Gy with vehicle treatment. The experiments were performed 2 times.

We also investigated whether the inhibition of ferroptosis restores macrophage phagocytosis. Ferrostatin-1 (Fer-1), a ferroptosis inhibitor known to suppress lipid peroxidation (21), was evaluated for its ability to protect macrophages from radiation-induced cell death and restoration of phagocytic function. Treatment of irradiated macrophages with Fer-1 (2 µM) attenuated lipid peroxidation (**Figures 6A, B**), and decreased LDH release compared to vehicle-treated macrophages (**Figure 6C**). Furthermore, Fer-1 treatment improved phagocytic function in irradiated macrophages, which correlated with restoring Rac-1 protein levels (**Figures 6D–F**). These findings confirm that ferroptosis contributes to radiation-induced macrophage phagocytic dysfunction as demonstrated by the improved phagocytosis following Fer-1 treatment.

## Discussion

Radiation exposure, whether from nuclear events, terrorist attacks, or medical procedures like cancer radiotherapy (3, 4), can induce ARS, characterized by hematopoietic stem and progenitor cell damage, leading to cytopenia and potentially multiple organ dysfunction (5, 6). Understanding the cellular and molecular mechanisms of radiation damage is essential for developing effective countermeasures and mitigating radiation injury.

**Figure 7:**
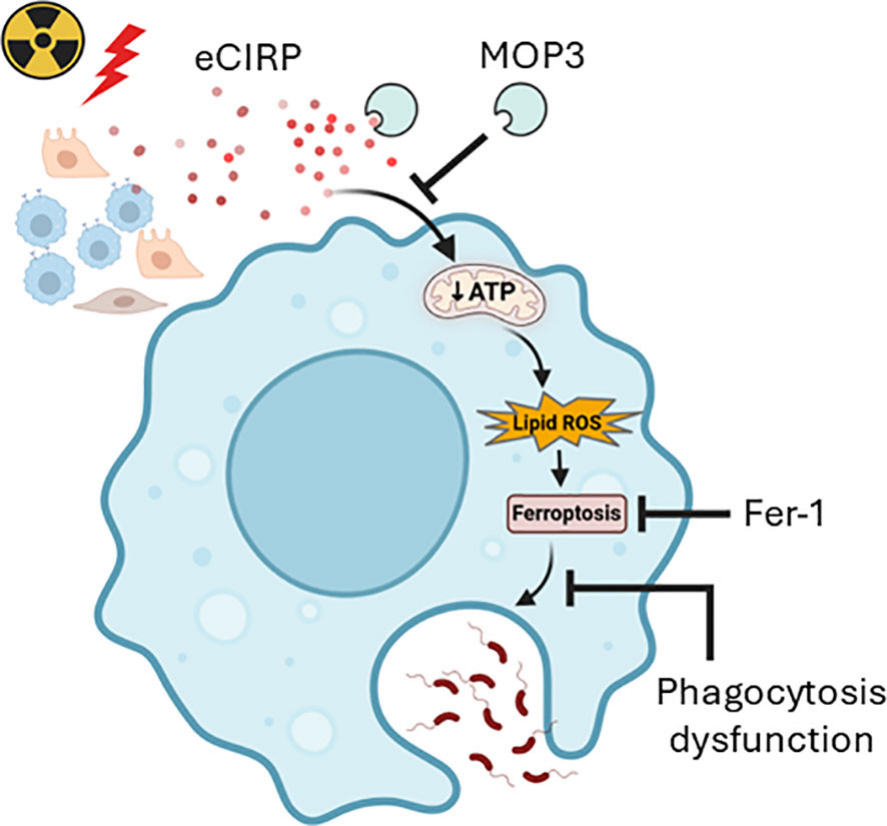
Graphical summary. Radiation-induced eCIRP release impairs mitochondria function and causes ferroptosis leading to macrophage phagocytotic dysfunction. Clearing eCIRP using a novel opsonic eCIRP inhibitor, MOP3 improved mitochondrial function, reduced ferroptosis, and restored macrophage phagocytosis after irradiation. While inhibiting ferroptosis also improved phagocytosis, it did not improve mitochondrial function, suggesting that eCIRP acts upstream in phagocytic dysfunction after irradiation. This schema was created using Biorender software.

While radiation-induced neutropenia increases susceptibility to infection, the relative radioresistance of macrophages and its role in innate immunity highlights their importance in post-radiation host defense (5, 8). However, the direct effects of radiation on macrophages and the underlying mechanisms remain poorly understood (8). The aim of the current study is to elucidate the impact of radiation on macrophage survival, viability, and phagocytotic function. Following a single dose of 10-Gy irradiation, macrophage phagocytotic function was significantly impaired. We observed ferroptosis occurring on Day 3 post-irradiation, increasing further by Day 5, as evidenced by increased lipid peroxidation and reduced GPX4 levels. This is supported by macrophage LDH release and reduced cell viability. We also revealed that radiation-induced eCIRP release caused mitochondrial dysfunction. The time-dependently released eCIRP post-irradiation positively correlated with the increase of ferroptosis and macrophage phagocytotic dysfunction. Our study clearly establishes that eCIRP directly causes mitochondrial dysfunction, although the precise underlying mechanism requires further investigation. This critical role of eCIRP in mitochondrial injury is substantiated by our finding that MOP3-mediated eCIRP clearance significantly improved mitochondrial function. While previous research has linked eCIRP to GPX4-mediated ferroptosis (22) and impaired macrophage phagocytosis in sepsis (23), the upstream drivers of these effects have remained elusive. We now demonstrate that eCIRP-induced mitochondrial dysfunction serves as a pivotal upstream pathway for both ferroptosis and subsequent phagocytic impairment, as evidenced by MOP3’s ability to restore mitochondrial function, reduce ferroptosis, and improve phagocytosis simultaneously. Furthermore, we show that ferroptosis-induced cell membrane damage negatively impacts phagocytosis, as confirmed by the improved phagocytic function upon Ferrostatin-1-mediated ferroptosis inhibition. Consequently, this paper is the first to identify eCIRP-induced mitochondrial dysfunction as a critical upstream event dictating ferroptosis and phagocytic dysfunction. Target eCIRP may represent an effective therapeutic approach for mitigating radiation-induce macrophage phagocytotic dysfunction (**Figure 7**).

Macrophages, crucial innate immune cells, are recruited to injury sites where they perform phagocytic functions (8). Previous research indicates that radiation dose differentially affects macrophage activation: low doses (< 2-Gy) induce pro-inflammatory activation, while higher doses (> 8-Gy) promote anti-inflammatory responses (8, 24). However, a study using cumulative ionizing radiation (10-Gy, delivered as 2-Gy/fraction/day for 5 days) on human monocyte-derived macrophages reported increased proinflammatory markers (CD80, CD86, HLA-DR) and decreased anti-inflammatory markers (CD163, IL-10) (24). Although this cumulative irradiation induced macrophage DNA damage and triggered a signaling response involving Checkpoint kinase 2 activation, no cleavage of caspase-3/-7 or PARP was observed, suggesting that apoptosis was not occurring in human macrophages after 10 Gy cumulative irradiation (24). In the current study, macrophages exposed to single 10-Gy irradiation dose showed no immediate cell death at 24 h post-irradiation based on LDH release and cell viability (data not shown) and showed no significant LDH release even at 48 h after irradiation. However, hallmarks of ferroptosis, including increased lipid peroxidation and decreased GPX4, emerged by Day 3 and intensified by Day 5. This delayed onset of cell death was further supported by increased LDH release and decreased cell viability on Days 3 and 5. The delayed occurrence of ferroptosis after irradiation suggests it is a secondary effect of initial radiation-induced cellular damage signals. To investigate the underlying mechanism, we considered eCIRP, previously shown to induce macrophage ferroptosis (22). Irradiated macrophages exhibited a significant increase in eCIRP release, which correlated strongly with the temporal profile of ferroptosis, linking radiation-induced eCIRP release to macrophage ferroptosis.

We then explored how eCIRP mediates ferroptosis. Mitochondria are vital for cellular energy production, signaling, and regulated cell death, particularly ferroptosis, by controlling ROS production, iron homeostasis, and lipid peroxidation (13, 20, 25). Our findings revealed impaired mitochondrial function by day 3 post-irradiation, characterized by reduced basal and maximal respiration and ATP production, which further declined by day 5. This mitochondrial dysfunction correlated with macrophage ferroptosis. Crucially, treating macrophages with eCIRP directly impaired mitochondrial function, confirming eCIRP’s role in mitochondrial injury. Finally, we utilized MOP3, an MFG-E8-derived oligopeptide known to bind eCIRP, reduce eCIRP-induced inflammation and mitigate sepsis and gut ischemia/reperfusion injury by enhancing eCIRP clearance (17, 18). MOP3 can strongly bind eCIRP to facilitate its internalization and lysosome-dependent degradation (17). Treating irradiated macrophages with MOP3 successfully reduced eCIRP levels, improved mitochondrial function, and inhibited ferroptosis. Collectively, our results demonstrate that eCIRP critically contributes to radiation-induced macrophage ferroptosis through mitochondrial impairment.

Ferroptosis is a distinct form of iron-dependent programmed cell death, differing from apoptosis, necrosis, and autophagy (9, 26). It is driven by accumulated iron (Fe^2+^ and Fe^3+^) that generates ROS through the Fenton reaction. Ferroptosis is fundamentally characterized by oxidative stress, lipid peroxidation, and the activity of ferroptosis defense pathways (27). ROS initiate lipid peroxidation in the presence of iron. Ferroptosis defense pathways, including GPX4, eliminate peroxidized lipids and protect cells from ferroptosis. Lipid peroxidation is a hallmark of ferroptosis, directly destroying cellular membranes and thereby causing cell death. The balance between factors promoting or inhibiting lipid peroxidation dictates cell fate (27). We observed that GPX4 protein expression was significantly decreased on day-3 and further decreased by day-5 post-irradiation, indicating a progressive loss of anti-lipid peroxidation and anti-ferroptotic defense in irradiated cells. While intracellular iron levels did not change on day-3, they significantly increased by day-5 post-irradiation (**Supplementary Figure 3**). This suggests that iron accumulation was not the sole contributor to ROS production in the earlier post-irradiation period. Thus, the progressive loss of GPX4 appears to play a critical role in radiation-induced ferroptosis. Morphologically, ferroptotic cells exhibit reduced mitochondrial volume, increased mitochondrial membrane density, loss of mitochondrial cristae, and plasma membrane rupture (9, 26). Our results demonstrate that radiation-induced mitochondria injury is associated with macrophage ferroptosis. We hypothesized that radiation-induced mitochondria injury by compromising mitochondrial energy production and losing membrane integrity in ferroptotic cells, would negatively affect macrophage phagocytic function. Our data confirmed this: irradiated macrophages exhibited reduced bacterial phagocytosis by day 3 post-irradiation, worsening by day 5. Phagocytosis is critically dependent on actin polymerization, which drives lamellipodia formation and plasma membrane ruffling for engulfment of target particles (23, 28). Rac1, a key regulator of this process, was significantly reduced in macrophages on day 3 and 5 post-irradiation, directly correlating with the observed phagocytic dysfunction. This finding aligns with our previous study showing eCIRP impairs macrophage phagocytosis through dysregulation of actin polymerization and reduced Rac-1 activation (23). Furthermore, other studies have linked radiation-induced phagocytosis dysfunction to decreased Rac1 and ARP2 levels (29). Thus, the loss of Rac1 and subsequent actin dysregulation are key contributors to post-irradiation phagocytic dysfunction. Mitochondrial stress and dysfunction initiate cellular responses that perturb organelle homeostasis and disrupt nuclear-mitochondrial gene expression coordination (19, 30). While mitochondrial ROS can attenuate cytosolic translation via ribosomal protein oxidation (30), further investigation is needed to elucidate how eCIRP-induced mitochondrial dysfunction specifically alters Rac1 and other protein expression levels after irradiation. To focus exclusively on the pure macrophage response and thereby avoid the complex cell-cell interactions inherent in *in vivo* studies, we did not include *in vivo* experiments in the current work. We acknowledge that this approach is a limitation of the present study.

In summary, we demonstrate that post-irradiation, macrophages undergo ferroptosis, which correlates with significant phagocytic dysfunction. Our research further identifies eCIRP release following radiation as a mediator of this ferroptosis. Critically, we elucidate how eCIRP-induced mitochondrial dysfunction converges with ferroptosis to impair macrophage phagocytosis. These findings suggest that targeting eCIRP could represent a novel therapeutic countermeasure against radiation-induced damage.

## Data Availability

The original contributions presented in the study are included in the article/**Supplementary Material**. Further inquiries can be directed to the corresponding authors.

## Supplementary material

Supplementary Figure 1 Peritoneal macrophages (PerM) were treated with rmCIRP (0.3 µg/ml) for 48 h and mitochondria function was assessed using Mito Stress Assay in a Seahorse XF Analyzer. **(A)** the real-time measurement of cellular oxygen consumption rate (OCR) indicated as kinetic is shown. **(B-D)** Basal and maximal respiration and ATP production are shown in bar diagrams. The data are expressed as the mean ± SE (n = 6/group). The control was set to 100 for normalization. The groups were compared by unpaired two tailed Student t-test. **p* < 0.05 vs. control group.

Supplementary Figure 2 Peritoneal macrophages were irradiated with a single dose of 10-Gy and the released eCIRP in the culture medium was measured by ELISA. Data are expressed as the mean ± SE (n = 4-5/group). The groups were compared by one-way ANOVA and the Tukey’s test. **p* < 0.05 vs. non-radiation control; ^#^*p* < 0.05 vs. day-2 post-irradiation;^!^*p* < 0.05 vs. day-3 post-irradiation.

Supplementary Figure 3 Peritoneal macrophages were irradiated with a single dose of 10-Gy and the intracellular iron levels were measured. The control was set to 1 for normalization. Data are expressed as the mean ± SE (n = 5-9/group). The groups were compared by one-way ANOVA and the Tukey’s test. **p* < 0.05 vs. non-radiation control; ^#^*p* < 0.05 vs. day-3 post-irradiation.
