# Supplementary figures and images for: Radiation-induced eCIRP causes macrophage phagocytic dysfunction via mitochondrial impairment and ferroptosis
Source: Front Immunol. 2025 Nov 20;16:1719613. doi: 10.3389/fimmu.2025.1719613 (PMC12675233; doi:10.3389/fimmu.2025.1719613)

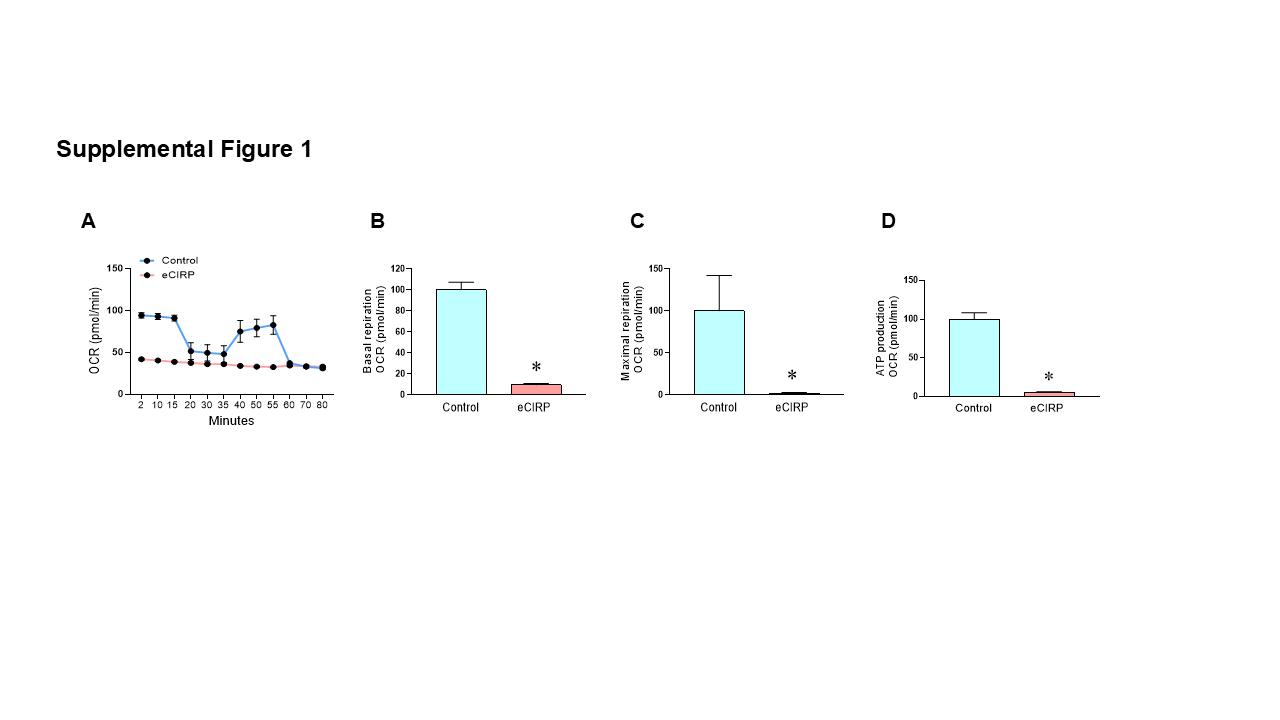

Supplement: Supplementary Figure 1 — Peritoneal macrophages (PerM) were treated with rmCIRP (0.3 µg/ml) for 48 h and mitochondria function was assessed using Mito Stress Assay in a Seahorse XF Analyzer. (A) the real-time measurement of cellular oxygen consumption rate (OCR) indicated as kinetic is shown. (B-D) Basal and maximal respiration and ATP production are shown in bar diagrams. The data are expressed as the mean ± SE (n = 6/group). The control was set to 100 for normalization. The groups were compared by unpaired two tailed Student t-test. *p < 0.05 vs. control group. [file Image1.tif]

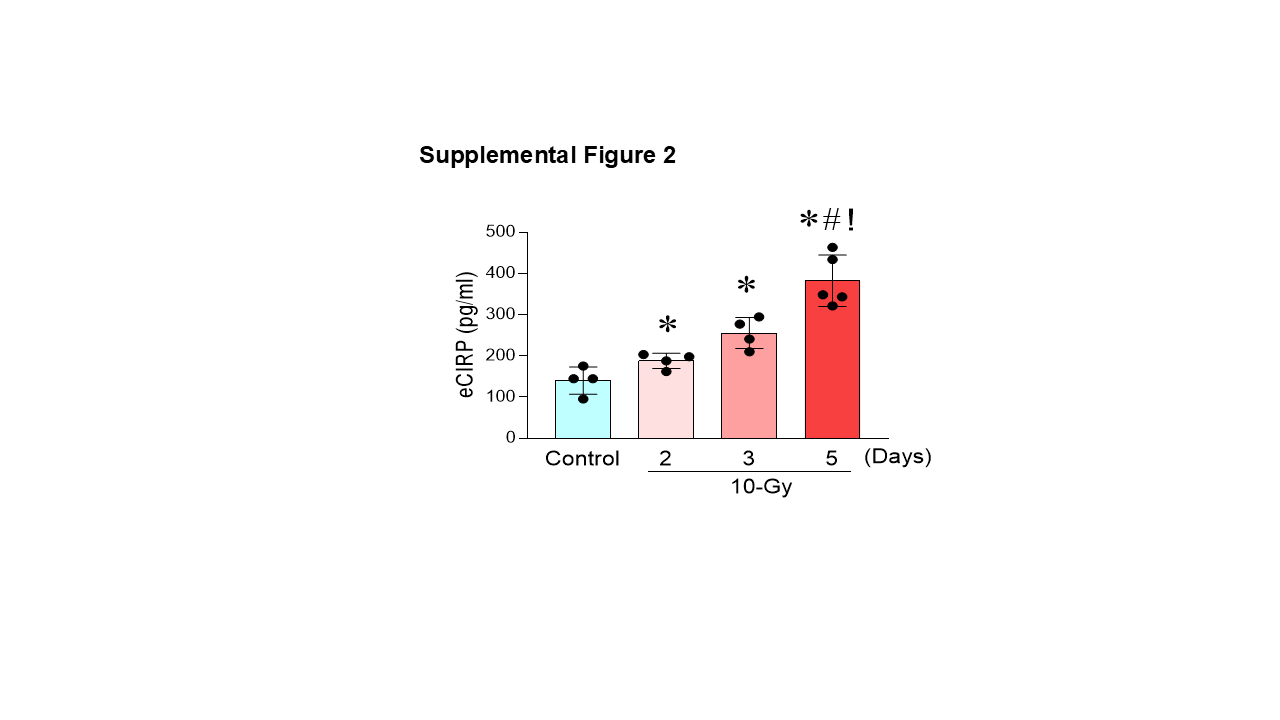

Supplement: Supplementary Figure 2 — Peritoneal macrophages were irradiated with a single dose of 10-Gy and the released eCIRP in the culture medium was measured by ELISA. Data are expressed as the mean ± SE (n = 4-5/group). The groups were compared by one-way ANOVA and the Tukey’s test. *p < 0.05 vs. non-radiation control; #p < 0.05 vs. day-2 post-irradiation;!p < 0.05 vs. day-3 post-irradiation. [file Image2.tif]

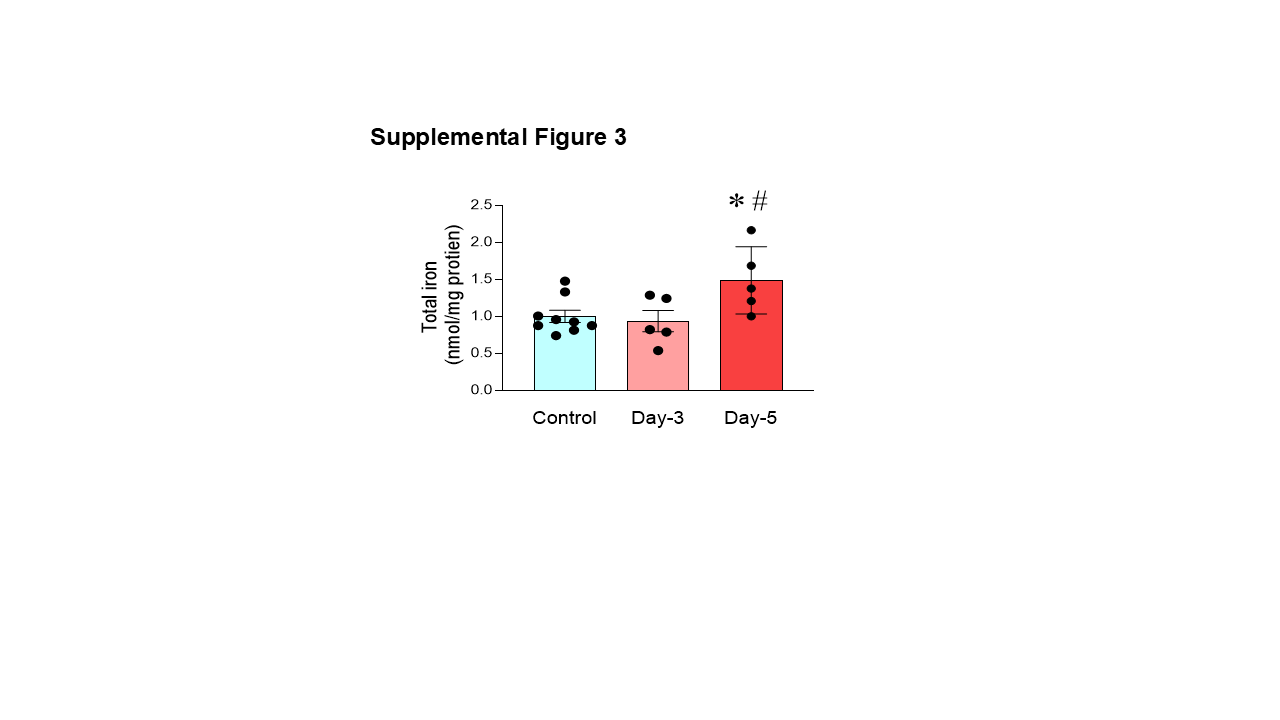

Supplement: Supplementary Figure 3 — Peritoneal macrophages were irradiated with a single dose of 10-Gy and the intracellular iron levels were measured. The control was set to 1 for normalization. Data are expressed as the mean ± SE (n = 5-9/group). The groups were compared by one-way ANOVA and the Tukey’s test. *p < 0.05 vs. non-radiation control; #p < 0.05 vs. day-3 post-irradiation. [file Image3.tif]
